# Associations of HALP score with serum prostate-specific antigen and mortality in middle-aged and elderly individuals without prostate cancer

**DOI:** 10.3389/fonc.2024.1419310

**Published:** 2024-09-20

**Authors:** Zhaoyang Chen, Yuanfeng Zhang, Mingjiang Dan, Xuwei Hong, Si Chen, Xiaojian Zhong

**Affiliations:** ^1^ Department of Urology, The Affiliated Shunde Hospital of Jinan University (The Second People’s Hospital of Shunde), Foshan, China; ^2^ Department of Urology, Shantou Central Hospital, Shantou, China; ^3^ Department of Urology, HuiYa Hospital of the First Affiliated Hospital, Sun Yat‐sen University, Huizhou, China; ^4^ Department of Urology, The First Affiliated Hospital of Shantou University Medical College, Shantou, Guangdong, China

**Keywords:** HALP score, prostate cancer, mortality, PSA, NHANES

## Abstract

**Background:**

The association between the Hemoglobin, Albumin, Lymphocyte, and Platelet (HALP) score and serum prostate-specific antigen (PSA) and all-cause mortality remains underexplored. We aimed to investigate the relationship between HALP score and these outcomes among middle-aged and elderly individuals without prostate cancer (PCa).

**Methods:**

This cross-sectional study included participants aged 40 years and older from National Health and Nutrition Examination Survey (NHANES) 2001–2010. HALP score was calculated using the formula: HALP score = (Hemoglobin × Albumin × Lymphocytes)/Platelets. High PSA level was defined as a percentage free PSA (%fPSA) less than or equal to 25% and a total PSA (tPSA) level equal to or higher than 4.0 ng/mL. Mortality data were obtained through December 30, 2019 by linking to the National Death Index.

**Results:**

Among 7,334 participants, 6,826 were classified as having low PSA level, while 508 were categorized as having high PSA level. Logistic regression revealed lower odds of high PSA level with higher HALP quartiles (*P*_trend_<0.001). Among 508 participants with high PSA level, over a median follow-up period of 10.13 years (IQR: 5.42-13.17 years), a total of 268 all-cause deaths were recorded. Cox regression analysis showed that participants in the highest HALP quartile had the lowest risk of all-cause mortality (HR = 0.527, 95% CI: 0.368-0.754) in participants with high PSA level. Restricted cubic spline analysis indicated a non-linear and negative correlation between HALP score and all-cause mortality, with an inflection point at 43.98 (*P* for non-linearity = 0.009). Random survival forest analysis ranked HALP score as the most significant predictor for all-cause mortality.

**Conclusion:**

Our study highlights that the HALP score the HALP score is associated with high PSA level and all-cause mortality among middle-aged and elderly individuals without PCa. Further research is warranted to validate these findings and elucidate underlying mechanisms.

## Introduction

Prostate cancer (PCa) stands as one of the most widespread malignancies impacting male health. In 2020, around 1.4 million new PCa cases were reported and an estimated 375,000 deaths caused by PCa in a quarter of the world’s countries (1). Managing patients with PCa remains a substantial challenge in contemporary medicine. Additionally, early detection and screening methods for PCa are not yet fully mature, leading to delayed diagnosis in some patients until the disease has progressed to advanced stages, thereby increasing the complexity of treatment. It is crucial to find out alternative methods to enhance the predictive accuracy of prognosis in PCa.

With the increasing acceptance of PCa screening, newer methodologies like magnetic resonance imaging (MRI) and biomarkers have been adopted to detect individuals likely to have prostate tumors. Serum prostate-specific antigen (PSA) levels are commonly employed in clinical diagnosis and has undoubtedly increased the identification of PCa lack of typical symptoms (2). Although significant advancement in diagnostic methods and treatment for PCa over the past decade, overall survival rates of patient with PCa still unsatisfactory (3). As a result, there is an urgent need to identify valuable biomarkers that can predict disease progression and survival. Currently, non-invasive identification of novel biomarkers in serum has increasingly captured the attention of researchers and clinicians alike, such as hemoglobin, platelet have been suggested to be prognosis indicators and included in prognosis models in some cancer studies (4, 5). It is worth noting that serum biomarker panel could provide a more comprehensive reflection of prognosis in PCa (6, 7). Biomarker from blood sample would be the most promising substrate for predicting these biomarkers in patients with PCa.

Both the inflammatory response and the nutrition status of the body play significant roles in the occurrence, progression, and prognosis of the cancer. Numerous inflammatory biomarkers derived from blood have been established as prognostic factors for PCa. In recent years, the Hemoglobin, Albumin, Lymphocyte, Platelet Score (HALP) has surfaced in the literature as a novel prognostic indicator utilized to predict various clinical outcomes across different types of cancers. As a novel immune-nutritional marker, the HALP integrates some routine serum markers that are easily obtained. The platelet and lymphocyte count could be representative indicators of immune status, while albumin and hemoglobin serve as nutritional status markers (8). The HALP was devised by Chen et al. to forecast the prognosis of gastric carcinoma in 2015 (9). Thereafter, the HALP score has demonstrated potential as a prognostic biomarker in different type of cancers, such as gastric carcinoma (9), colorectal cancer (10), lung cancer (11), bladder cancer (12), cervical cancer (13), as well as PCa (14). Besides, recent studies have proved that HALP score was a stronger predictor of outcomes in PCa patients compared to other biomarkers like Neutrophil-Lymphocyte Ratio (NLR) and Platelet-Lymphocyte Ratio (PLR) (14). Although previous research has reported the significant association between high NLR and PLR scores and an adverse prognosis in PCa patients (15, 16). In another study, it was discovered that the HALP could be used to differentiate men with benign prostate hyperplasia (BPH) and PCa (17). As a fast, effective and cheap biomarker, the HALP score could help clinicians to detect the risk of PCa and make more precise decisions on the patients with PCa. However, these studies did not assess overall survival or have enough long follow-up period.

The relationship between the HALP score, serum PSA, and all-cause mortality has not been thoroughly investigated. Therefore, to examine this association, we utilized data from the National Health and Nutrition Examination Survey (NHANES), which is representative of the U.S. population. We conducted a cohort study to explore the associations between the HALP score and serum PSA as well as survival outcomes among middle-aged and elderly individuals without PCa.

## Materials and methods

### Study population

NHANES is a comprehensive, multi-stage cross-sectional study established by the National Center for Health Statistics (NCHS) with the primary objective of assessing the health and nutritional status of both adults and children across the United States (https://www.cdc.gov/nchs/nhanes/index.htm). This survey serves to ascertain disease prevalence and its etiology, explore the intricate relationship between nutrition and health promotion, and inform the development of efficacious public health policies. The research protocols received approval from the NCHS Research Ethics Review Board, with all participants providing written informed consent.

Participants eligible for PSA testing were male individuals aged 40 years and older. Participants were excluded from PSA testing if they reported current prostate gland infection or inflammation, had undergone a rectal examination within the past week, prostate biopsy within the past month, or cystoscopy within the past month. These conditions were identified using self-reported data from the NHANES survey. They were deemed potential factors affecting PSA levels and were thus considered exclusion criteria for the study.

A total of 8,858 participants aged 40 years and older from NHANES 2001-2010 were initially included in the study. After excluding individuals with missing data for HALP calculation (n=891), those affecting PSA factors (n=119), participants diagnosed with PCa (n=333), and those lacking data on serum PSA testing (n=171), the final enrolled sample comprised 7,344 participants without PCa. During follow-up and eligibility assessment, an additional 10 participants were excluded, leaving a final analysis sample of 7,334 participants. Among these participants, 6,826 were classified as having low PSA level, while 508 were categorized as having high PSA level (**Supplementary Figure S1**).

### Assessment of HALP

The complete blood count (CBC) parameters, including hemoglobin, Lymphocyte and platelet count, were generated using the Beckman Coulter MAXM instrument. Hemoglobin concentration was measured using the Beckman Coulter method, which employs automatic dilution and mixing devices along with single-beam photometry for accurate hemoglobinometry. Lymphocyte count and platelet count were also derived from CBC parameters using VCS technology for WBC differential analysis. Albumin concentration was determined utilizing the DcX800 method, a bichromatic digital endpoint method. In this method, albumin forms a complex with Bromcresol Purple (BCP) reagent, and the change in absorbance at 600 nm is monitored, providing a direct measure of albumin concentration in the sample.

The HALP score was calculated as per the established formula utilizing laboratory data obtained from blood specimens collected at NHANES Mobile Examination Centers (MECs) and processed using standardized methodologies. The HALP score was computed using the formula: hemoglobin (g/L) × albumin (g/L) × lymphocytes (/L)/platelets (/L).

### Assessment of serum PSA levels

PSA levels were assessed through separate assays for free and total PSA (fPSA and tPSA). The fPSA assay utilized a two-site immunoenzymatic “sandwich” technique, employing monoclonal anti-fPSA antibodies conjugated to alkaline phosphatase in a reaction vessel. Paramagnetic particles coated with another anti-fPSA antibody facilitated fPSA binding, with chemiluminescent substrate addition producing light proportional to fPSA concentration. tPSA values were obtained using a similar assay setup on the Beckman Access platform, where tPSA bound to immobilized monoclonal anti-PSA. Additionally, the percentage of fPSA (%fPSA) was calculated by dividing fPSA by tPSA and rounding to the nearest whole number. Detailed laboratory methods are accessible on the NHANES website. Detailed laboratory method descriptions are available on the NHANES website at https://wwwn.cdc.gov/Nchs/Nhanes/2009-2010/PSA_F.htm.

As defined in previous studies (18), individuals with %fPSA greater than 25% and tPSA level less than 4.0 ng/ml were categorized as low PSA level, while those with %fPSA less than or equal to 25% and tPSA level equal to or higher than 4.0 ng/ml were classified as high PSA level.

### Assessment of mortality

The assessment of mortality within the NHANES database involves a comprehensive procedure aimed at linking participant data with national mortality databases, notably the National Death Index (NDI). This linkage ensures the accurate determination of participant deaths, thus facilitating meticulous maintenance of mortality records within the study cohort. The follow-up period extends until December 31, 2019, allowing for a thorough examination of long-term mortality outcomes.

### Other covariates

NHANES is a comprehensive data collection initiative that integrates interviews and physical examinations to gather extensive information. In our study, we meticulously controlled for various potential confounders to ensure the robustness of our findings. These included demographic factors such as age (grouped as 40-59 years or ≥60 years), and race (classified as Non-Hispanic White, Non-Hispanic Black, or Other). Furthermore, socioeconomic status was accounted for using the family income-to-poverty ratio (PIR), with categories defined as ≤1.0, 1.1–3.0, or >3.0, alongside education level (below high school, high school, or above high school). Lifestyle factors such as smoking status (never smoker, former smoker, or current smoker) and drinking status (nondrinker, low-to-moderate drinker, or heavy drinker) were also considered. Moreover, energy intake levels were stratified into quartiles to control for dietary variations. Physical activity levels were categorized as inactive, insufficiently active, or active. Furthermore, dietary quality was assessed using the Healthy Eating Index (HEI), divided into quartiles, while comorbidity burden was evaluated using the Charlson Comorbidity Index (CCI), considered as a continuous variable. For comprehensive definitions of family PIR, smoking status, drinking status, physical activity levels, HEI, and CCI, please refer to the methodology section of the **Supplementary Materials**.

### Statistical analysis

To ensure nationally representative estimates, we followed guidelines from the National Center for Health Statistics, incorporating primary sampling units, sample weights, and strata during data analysis. Weighted analyses were conducted using the “survey” package in R. Continuous variables were presented as mean (standard error [SE]), with normally distributed ones subjected to Student’s t-test or One-Way ANOVA. Categorical variables were expressed as numbers (percentages). Chi-square testing assessed unordered categorical variables, while the Kruskal-Wallis H test evaluated ordered categorical variables. Missing data were imputed using the “mice” package and the random forest algorithm.

Linear regression analyzed the association between HALP score quartiles and serum PSA levels, reporting β and 95% CIs. Logistic regression examined the association between HALP score quartiles and high PSA level, reporting odds ratios (ORs) and 95% CIs. Restricted cubic spline curves explored potential non-linear links between HALP score and high PSA level.

Survival analysis utilized the log-rank test with Kaplan-Meier survival curves, and COX proportional hazard regression assessed the association of HALP score with all-cause mortality, reporting hazard ratios (HRs) and 95% CIs. Assumptions were checked using Schoenfeld residuals, and multicollinearity was examined using variance inflation factors (VIF). Multivariable Cox regression models with restricted cubic splines explored potential non-linear links between HALP score and mortality in participants with high PSA level.

Time-dependent receiver operating characteristic (ROC) curves evaluated the predictive capability of HALP score and its components for mortality, reporting area under the curve (AUC). Spearman’s correlation analysis calculated correlation coefficients between HALP score and its components. Significant predictive features for mortality were identified using the random survival forest (RSF) model, providing feature importance measures. Statistical analyses were performed using R (version 4.3.2), with significance set at *P* < 0.05.

## Results

### Baseline characteristics of the participants

**Table 1** outlines baseline characteristics of 7,334 participants aged 40 years and older from NHANES 2001–2010, categorized by HALP score quartiles. The majority of participants (67.56%) fell within the 40-59 age range, with the remaining 32.44% aged 60 and above. Significant differences were observed across HALP quartiles for demographic variables such as age, race/ethnicity, living status, and education level (*P* < 0.001). Moreover, family PIR, smoking and drinking status, BMI, physical activity levels, HEI-2015 score, CCI, hemoglobin, albumin, lymphocyte, and platelet counts also showed significant variations among HALP quartiles (all *P* < 0.001). Analysis revealed significant differences in PSA distribution across HALP quartiles. Participants in the highest HALP quartile had lower median tPSA (0.83 ng/mL, p < 0.001) and fPSA levels (0.25 ng/mL, *P* < 0.001) compared to lower quartiles. %fPSA showed a slight but significant difference across quartiles (*P* = 0.017), with the highest quartile at 30.00%. PSA classification varied significantly across quartiles (*P* < 0.001), with lower HALP quartiles having a higher proportion of participants with high PSA level (7.81% in the lowest quartile vs. 3.03% in the highest quartile).

**Table 1:** Baseline characteristics of middle-aged and elderly individuals without prostate cancer according to quartiles of HALP in NHANES 2001–2010.

Characteristics	Total	Quartiles of HALP				*P* value
		<39.40	39.41-52.03	52.04-67.86	>67.86	
Participants	7334	1834	1833	1833	1834	
Age, %						<0.001
40-59 years	3730(67.56)	736(57.02)	964(69.52)	992(70.80)	1038(71.93)	
≥60 years	3604(32.44)	1098(42.98)	869(30.48)	841(29.20)	796(28.07)	
Race/ethnicity, %						<0.001
Non-Hispanic White	4012(77.10)	1053(77.78)	1040(79.37)	982(77.50)	937(73.59)	
Non-Hispanic Black	1332(8.85)	402(11.12)	319(8.05)	296(7.59)	315(8.89)	
Other race	1990(14.05)	379(11.10)	474(12.59)	555(14.90)	582(17.52)	
Living status, %						0.150
Alone	1997(23.53)	556(25.30)	449(22.44)	480(21.69)	512(24.99)	
With partners	5337(76.47)	1278(74.70)	1384(77.56)	1353(78.31)	1322(75.01)	
Education level, %						0.002
Below high school	2310(18.56)	569(18.44)	515(15.83)	591(18.63)	635(21.53)	
High school	1699(25.24)	412(24.31)	449(26.10)	409(23.66)	429(26.84)	
Above high school	3325(56.20)	853(57.25)	869(58.07)	833(57.70)	770(51.63)	
Family PIR, %						0.007
≤1.0	1162(9.29)	279(9.13)	263(8.28)	285(8.59)	335(11.26)	
1.1–3.0	2992(31.26)	764(32.76)	730(30.00)	747(29.26)	751(33.31)	
>3.0	3180(59.45)	791(58.11)	840(61.72)	801(62.15)	748(55.43)	
Smoking status, %						<0.001
Never smoker	2752(41.10)	719(42.96)	735(45.41)	690(40.68)	608(35.15)	
Former smoker	2893(36.60)	825(43.34)	733(36.13)	713(36.08)	622(31.35)	
Current smoker	1689(22.30)	290(13.70)	365(18.46)	430(23.25)	604(33.49)	
Drinking status, %						< 0.001
Nondrinker	1001(12.21)	308(16.00)	242(11.61)	233(11.14)	218(10.43)	
Low-to-moderate drinker	5550(76.40)	1353(73.45)	1379(75.52)	1407(78.28)	1411(78.16)	
Heavy drinker	783(11.39)	173(10.55)	212(12.87)	193(10.57)	205(11.41)	
Body mass index, %						< 0.001
<25.0 kg/m^2^	1711(21.38)	541(26.93)	442(21.79)	376(19.44)	352(17.76)	
25.0-29.9 kg/m^2^	3095(42.84)	735(40.73)	774(42.41)	797(43.81)	789(44.27)	
>29.9 kg/m^2^	2528(35.78)	558(32.35)	617(35.81)	660(36.75)	693(37.97)	
Physical activity, %						0.001
Inactive	1981(19.91)	577(24.42)	466(17.77)	450(18.05)	488(19.95)	
Insufficiently active	2921(45.75)	703(43.93)	757(48.06)	746(46.40)	715(44.28)	
Active	2432(34.34)	554(31.65)	610(34.18)	637(35.55)	631(35.77)	
HEI-2015 score	50.12(41.49,59.37)	51.15(42.34,61.36)	49.65(40.91,58.94)	50.21(42.15,59.39)	49.73(40.74,58.11)	0.003
CCI	1.00(0.02)	1.20(0.05)	0.91(0.04)	0.91(0.04)	1.01(0.04)	<0.001
tPSA, ng/mL	0.90(0.54,1.60)	1.00(0.60,1.86)	0.86(0.50,1.53)	0.89(0.54,1.53)	0.83(0.50,1.48)	<0.001
fPSA, ng/mL	0.26(0.17,0.42)	0.29(0.18,0.48)	0.26(0.16,0.42)	0.26(0.17,0.41)	0.25(0.16,0.39)	<0.001
%fPSA, %	30.0(22.0,38.0)	29.0(21.0,37.0)	29.0(22.0,39.0)	30.0(22.0,38.0)	30.0(22.0,40.0)	0.017
Hemoglobin, g/dL	15.2(14.5,16.0)	14.7(13.8,15.4)	15.1(14.5,15.9)	15.4(14.8,16.0)	15.6(14.9,16.4)	<0.001
Albumin, g/L	43.0(41.0,45.0)	42.0(40.0,44.0)	43.0(41.0,45.0)	43.0(42.0,45.0)	44.0(42.0,46.0)	<0.001
Lymphocyte, 103/μL	1.90(1.50,2.40)	1.40(1.10,1.60)	1.70(1.50,2.00)	2.10(1.80,2.40)	2.60(2.20,3.00)	<0.001
Platelet, 103/μL	239.0(203.0,280.0)	272.0(232.0,316.0)	246.0(214.0,286.0)	234.0(204.0,272.0)	210.0(179.0,243.0)	<0.001
PSA levels, %						<0.001
Low level	6826(95.13)	1640(92.19)	1704(94.64)	1739(96.53)	1743(96.97)	
High level	508(4.87)	194(7.81)	129(5.36)	94(3.47)	91(3.03)	
All-cause mortality, %						<0.001
No	5007(77.72)	1043(68.14)	1306(80.81)	1341(81.48)	1317(79.43)	
Yes	2327(22.29)	791(31.86)	527(19.20)	492(18.53)	517(20.57)	
Follow-up time, years	12.50(10.17,15.50)	12.33(9.42,15.42)	13.08(10.58,15.75)	12.67(10.33,15.50)	12.25(10.17,15.33)	<0.001

PIR, poverty income ratio; PSA, prostate-specific antigen; tPSA, total prostate specific antigen; fPSA, free prostate specific antigen; %fPSA, percent free prostate specific antigen; HEI-2015, Healthy Eating Index 2015; CCI, Charlson Comorbidity Index; HALP, hemoglobin, albumin, lymphocyte and platelet.

Normally distributed continuous variables are described as means ± SEs, and continuous variables without a normal distribution are presented as medians [interquartile ranges]. Categorical variables are presented as numbers (percentages). N reflect the study sample while percentages reflect the survey-weighted data.

**Supplementary Table S1** presents baseline characteristics of participants aged 40 years and older from NHANES 2001–2010, stratified by high and low PSA level. Notably, the median HALP score was lower in the high PSA level group (45.48, 32.72–57.13) compared to the low PSA level group (52.80, 40.67–67.99).

### The relationship between HALP score and high PSA level

**Supplementary Table S2** presents the results of linear regression analysis examining the association between HALP score and serum PSA levels among middle-aged and elderly individuals without PCa, categorized by quartiles of HALP score. For tPSA, there was a significant inverse association with increasing quartiles of HALP score in both crude and adjusted models (*P* trend < 0.001 for all models). Similar trends were observed for fPSA, with significant inverse associations across quartiles of HALP score in all models (P trend < 0.001 for all models). Additionally, %fPSA showed a significant positive association with increasing quartiles of HALP score in all models (*P* trend < 0.001 for all models). In Model 2, which was adjusted for age, race/ethnicity, living status, education level, family PIR, BMI, drinking status, smoking status, physical activity, HEI, and CCI, the associations remained statistically significant for all three PSA parameters (%fPSA, fPSA, and tPSA).

**Table 2** presents the results of logistic regression analysis investigating the association between HALP levels and PSA classification among middle-aged and elderly individuals without PCa. In the crude model, higher quartiles of HALP levels were associated with significantly lower odds of high PSA level compared to the lowest quartile (*P*_trend_ < 0.001). After adjusting for demographic factors (Model 1) and additional lifestyle and health-related variables (Model 2), the inverse association persisted, with higher HALP quartiles associated with reduced odds of high PSA level (*P*_trend_ < 0.001 for both models). Specifically, compared to the lowest quartile of HALP score, the ORs for high PSA level in the second, third, and fourth quartiles of HALP score are 0.850 (95% CI: 0.615-1.17), 0.552 (95% CI: 0.397-0.767), and 0.485 (95% CI: 0.349-0.674), respectively. Furthermore, RCS analysis indicated a linear correlation between HALP score and serum PSA levels (tPSA, fPSA, %fPSA), and high PSA level (all *P* for non-linearity > 0.05, **Supplementary Figure S2**).

**Table 2:** Logistic regression analysis of HALP with the high PSA level among middle-aged and elderly individuals without prostate cancer in NHANES 2001–2010.

	Quartiles of HALP levels				*P*_trend_
	<39.40	39.41-52.03	52.04-67.86	>67.86	
Crude	1 [Reference]	0.668(0.495,0.902)	0.424(0.307,0.587)	0.369(0.261,0.522)	<0.001
Model 1	1 [Reference]	0.830(0.613,1.125)	0.531(0.386,0.732)	0.470(0.331,0.667)	<0.001
Model 2	1 [Reference]	0.850(0.615,1.175)	0.552(0.397,0.767)	0.485(0.349,0.674)	<0.001

PSA, prostate-specific antigen; HEI-2015, Healthy Eating Index 2015; CCI, Charlson Comorbidity Index; HALP, hemoglobin, albumin, lymphocyte and platelet.

Data are presented as OR (95% CI) unless indicated otherwise. Model 1: Adjusted for age (40-59, or ≥60 years), and race/ethnicity (non-Hispanic White, non-Hispanic Black or other race); Model 2: Model 1 + living status (with partners, or alone), education level (below high school, high school, or above high school), family PIR (<1.0, or ≥1.0), BMI (<25.0, 25.0-29.9, or >29.9 kg/m^2^), drinking status (nondrinker, low-to-moderate drinker, or heavy drinker), smoking status (never smoker, former smoker, or current smoker), physical activity (inactive, insufficiently active, or active), HEI (in quartiles), and CCI (continous).

### Association of high PSA level with all-cause mortality

**Figure 1:**
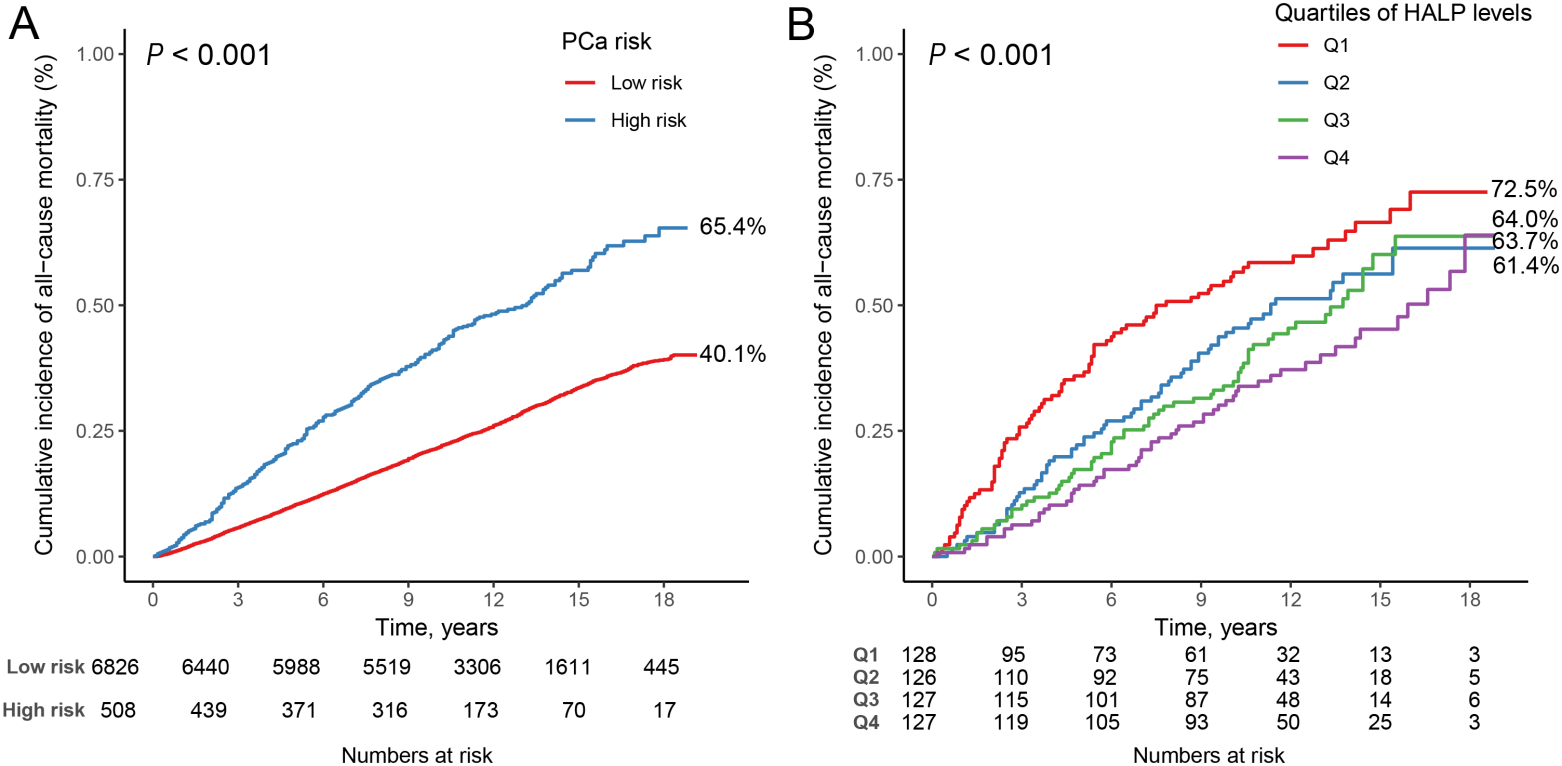
Kaplan-Meier survival curves for high PSA level, and HALP score, and all-cause mortality. **(A)** high PSA level and all-cause mortality in participants with men 40 years and older; **(B)** HALP score and all-cause mortality in participants with high PSA level.

A total of 2,327 all-cause deaths were documented among 7,334 male participants aged 40 years and older without PCa, with a median follow-up period of 11.67 years (IQR: 9.42-14.67 years). The Kaplan-Meier survival curves illustrated the association between high PSA level and all-cause mortality (**Figure 1A**). Notably, individuals classified with a high PSA level demonstrated a significantly elevated risk of all-cause mortality compared to those categorized with a low PSA level (log-rank *P* < 0.001). COX regression analysis further revealed that participants with high PSA level had a 35% increased risk of mortality compared with those at low PSA level (HR = 1.347, 95% CI: 1.183-1.534, *P* < 0.001).

### Association of HALP score with all-cause mortality

**Supplementary Table S3** presents the baseline characteristics of the 508 participants with high PSA level categorized by all-cause mortality. Over a median follow-up period of 10.13 years (IQR: 5.42-13.17 years), a total of 268 all-cause deaths were recorded. HALP score exhibited significant differences between the groups (*P* = 0.015), with the median HALP score being lower among individuals who experienced all-cause mortality (43.40, IQR: 30.45-54.02) compared to those who did not (47.16, IQR: 35.50-60.17). The Kaplan-Meier survival curves demonstrated the association between HALP score quartiles and all-cause mortality (**Figure 1B**). It is evident from the curves that participants in the Q4 group exhibited notably higher mortality compared to individuals in the other three quartile groups (log-rank *P*<0.001).

**Figure 2:**
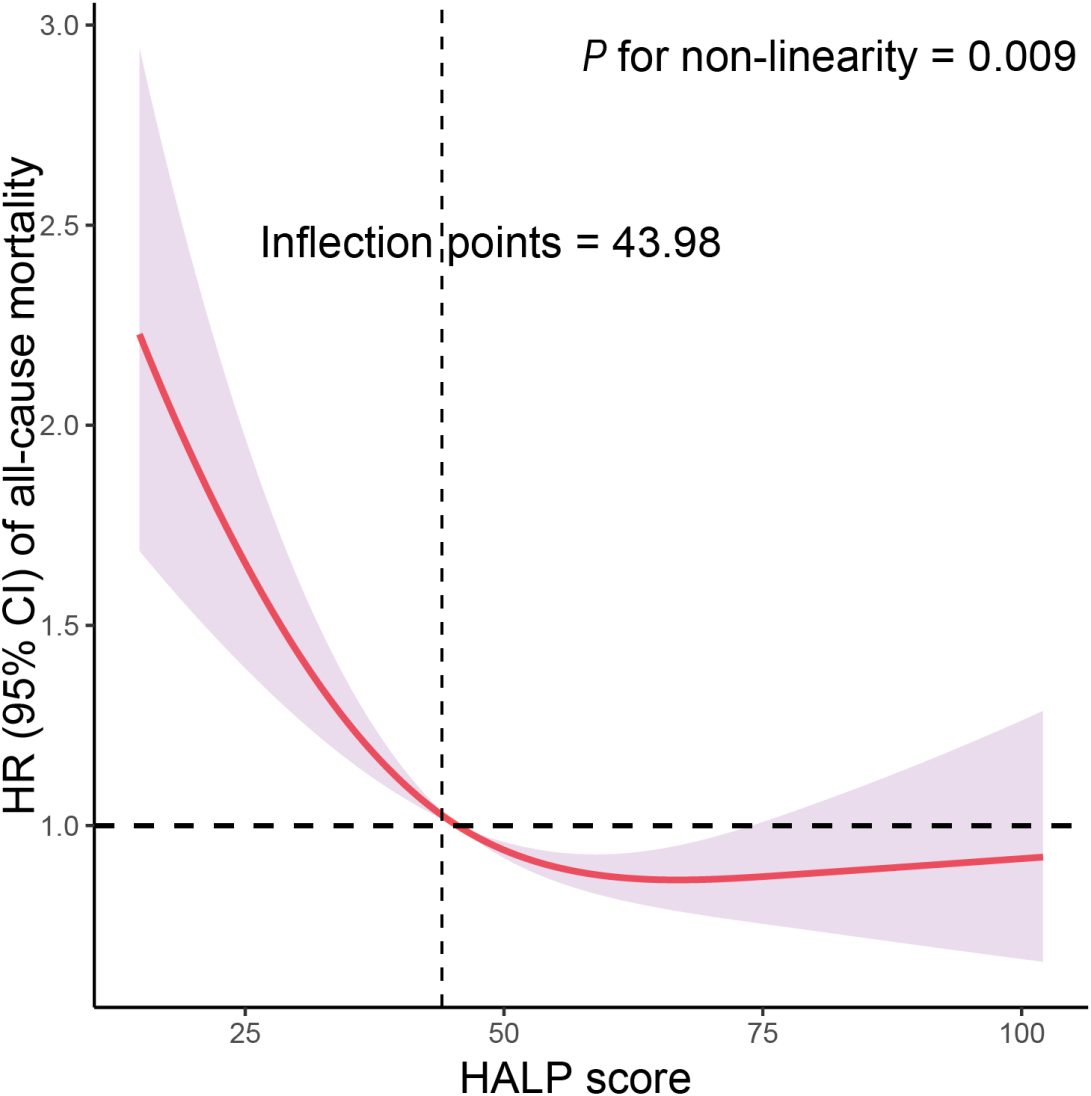
Restricted cubic spline (RCS) analysis with multivariate-adjusted associations of HALP score with all-cause mortality among participants with high PSA level in NHANES 2001–2010. Models are adjusted for age (40-59, or ≥60 years), race/ethnicity (non-Hispanic White, non-Hispanic Black or other race), living status (with partners, or alone), education level (below high school, high school, or above high school), family PIR (<1.0, or ≥1.0), BMI (<25.0, 25.0-29.9, or >29.9 kg/m2), drinking status (nondrinker, low-to-moderate drinker, or heavy drinker), smoking status (never smoker, former smoker, or current smoker), physical activity (inactive, insufficiently active, or active), HEI (in quartiles), and CCI (continuous).

**Table 3** displays the results of COX regression analysis investigating the association between HALP score quartiles and all-cause mortality stratified by high or low PSA level. These results indicate that compared to the reference group, participants in the higher HALP score quartiles had significantly lower risks of all-cause mortality. Specifically, after adjusting for various confounding factors, participants with high PSA level in the highest HALP quartile exhibited the lowest risk of all-cause mortality, with a HR of 0.527 (95% CI: 0.368-0.754, *P*<0.001); and participants with low PSA level in the highest HALP quartile were associated with a reduced risk of all-cause mortality, with an HR of 0.806 (95% CI: 0.714-0.909, *P*<0.001) compared to those in the lowest HALP quartile. The interaction between PSA levels and HALP scores in relation to all-cause mortality was not significant (*P* for interaction = 0.586), indicating that the association between HALP score and mortality did not significantly differ between individuals with high and low PSA levels. Furthermore, RCS analysis indicated a non-linear and negative correlation between HALP score and all-cause mortality, with an inflection point at 43.98 (*P* for non-linearity = 0.009, **Figure 2**). As HALP score increased, the HR for all-cause mortality declined rapidly until reaching the inflection point, after which it stabilized, indicating a plateau phase with no further significant increase in mortality risk associated with higher HALP scores. Furthermore, we further conducted a sensitivity analysis to examine the association of the HALP score with all-cause mortality after excluding participants who had a history of cancer at baseline. The results of this analysis, presented in **Supplementary Table S4**, indicate that the findings are consistent with our primary analysis, showing no significant changes in the association between the HALP score and all-cause mortality.

**Table 3:** COX regression analysis of HALP score with all-cause mortality stratified by high or low PSA level among middle-aged and elderly individuals without prostate cancer in NHANES 2001–2010.

	Crude		Model 1		Model 2	
	HR (95% CI)	*P* value	HR (95% CI)	*P* value	HR (95% CI)	*P* value
High PSA level (n = 508)						
Quartiles of HALP levels						
Q1	1 [Reference]		1 [Reference]		1 [Reference]	
Q2	0.689(0.499,0.952)	0.024	0.677(0.489,0.937)	0.019	0.766(0.548,1.071)	0.119
Q3	0.615(0.443,0.853)	0.004	0.600(0.431,0.834)	0.002	0.693(0.487,0.987)	0.042
Q4	0.491(0.348,0.692)	<0.001	0.551(0.391,0.776)	<0.001	0.527(0.368,0.754)	<0.001
*P* for trend		<0.001		<0.001		<0.001
Low PSA level (n = 6,826)						
Quartiles of HALP levels						
Q1	1 [Reference]		1 [Reference]		1 [Reference]	
Q2	0.604(0.537,0.679)	<0.001	0.705(0.627,0.794)	<0.001	0.753(0.669,0.848)	<0.001
Q3	0.576(0.511,0.649)	<0.001	0.691(0.613,0.779)	<0.001	0.742(0.656,0.838)	<0.001
Q4	0.640(0.569,0.719)	<0.001	0.795(0.706,0.895)	<0.001	0.806(0.714,0.909)	<0.001
*P* for trend		<0.001		<0.001		<0.001
*P* for interaction		0.540		0.813		0.586

PSA, prostate-specific antigen; HEI-2015, Healthy Eating Index 2015; CCI, Charlson Comorbidity Index; HALP, hemoglobin, albumin, lymphocyte and platelet.

Data are presented as OR (95% CI) unless indicated otherwise. Model 1: Adjusted for age (40-59, or ≥60 years), and race/ethnicity (non-Hispanic White, non-Hispanic Black or other race); Model 2: Model 1 + living status (with partners, or alone), education level (below high school, high school, or above high school), family PIR (<1.0, or ≥1.0), BMI (<25.0, 25.0-29.9, or >29.9 kg/m^2^), drinking status (nondrinker, low-to-moderate drinker, or heavy drinker), smoking status (never smoker, former smoker, or current smoker), physical activity (inactive, insufficiently active, or active), HEI (in quartiles), and CCI (continous).

**Supplementary Figure S3** displays Kaplan-Meier survival curves illustrating the relationship between quartiles of HALP components and all-cause mortality among participants with high PSA level. The results showed that statistically significant differences in mortality were observed for hemoglobin and lymphocyte count quartiles, while no significant differences were found for serum albumin and platelet count quartiles. COX regression analysis indicated that higher quartiles of hemoglobin and lymphocyte count were associated with reduced mortality hazards, whereas no significant associations were observed for albumin and platelet count after adjusting for covariates (**Supplementary Table S4**). Additionally, RCS analysis revealed a non-linear and negative correlation between hemoglobin and lymphocyte count and all-cause mortality (all *P* for non-linearity < 0.05), with inflection points at 14.34 and 1.61, respectively (**Supplementary Figures S4A**, **S4C**). However, a linear correlation was observed between serum albumin and platelet count and all-cause mortality (all *P* for non-linearity > 0.05, **Supplementary Figures S4B**, **S4D**).

### Prediction of mortality by HALP score and its components

**Figure 3:**
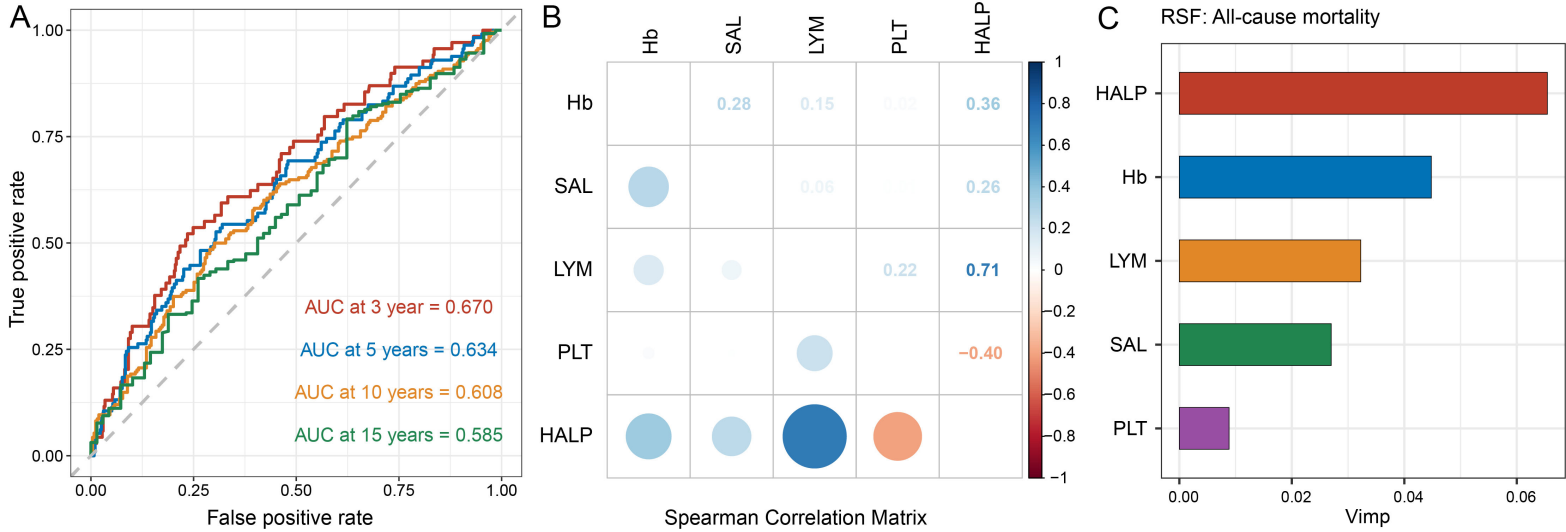
Capability of HALP score and its components to predict mortality. **(A)** Time-dependent ROC curves were used to assess the predictive capability of HALP score for 3-, 5-, 10-, and 15-year all-cause mortality in participants with high PSA level; **(B)** Spearman's correlation analysis was used to calculate the correlation coefficients among HALP score and its components; **(C)** Ranking the significance of the HALP score and its components in predicting all-cause mortality using random survival forest analysis.

ROC curve analysis revealed that HALP score exhibited the highest AUC for predicting all-cause mortality compared to individual components, including Hb, SAL, LYM, and PLT (**Supplementary Figure S5**). Specifically, HALP score demonstrated AUC values of 0.670, 0.634, 0.608, and 0.585 at 3, 5, 10, and 15 years, respectively (**Figure 3A**). **Figure 3B** illustrates the correlation coefficients matrix among HALP score and its components, indicating a strong positive correlation between HALP score and LYM (r=0.71, *P* < 0.001), followed by PLT (r=-0.40, *P* < 0.001), Hb (r=0.36, *P* < 0.001), and SAL (r=0.26, *P* < 0.001). Additionally, the RSF analysis ranked the importance of HALP score and its components in predicting mortality, with HALP score identified as the most significant predictor for all-cause mortality (**Figure 3C**).

## Discussion

Based on the NHANES database, we investigated the correlations between HALP score and different levels of PSA, as well as all-cause mortality, among middle-aged and elderly individuals without PCa. In our study, we have demonstrated that there was a protective association between higher levels of HALP and the PSA level. Individuals with high PSA level exhibited a significantly heightened risk of all-cause mortality when compared to those with low PSA level. Individuals with higher quartiles of HALP scores exhibited significantly diminished risks of all-cause mortality. There was a non-linear and inverse correlation between HALP score and all-cause mortality. Moreover, the HALP score was considered as the most significant predictor for all-cause mortality compared to its components among individuals with high PSA level.

PCa is the second most common cancer and one of the leading causes of cancer-related death in the male population (19). The primary method for detecting PCa at its early stages typically involves the widely utilized PSA blood test, followed by biopsy for confirmation of diagnosis (20). For example, a distinctive trait of PCa is often characterized by a reduced ratio of fPSA to tPSA (20). Elevated serum PSA levels do not always indicate the presence of these cancers. Other factors, such as BPH or inflammation, can also lead to increased serum PSA levels (21). PSA is not specific on its own and should be interpreted alongside other clinical factors. Our study population specifically excluded factors that could influence serum PSA levels, such as prostatitis, current inflammation. PSA level can reflect cancer behavior and possible progression of disease, may not fully represent the actual prognosis of PCa. Moreover, there was a significant inverse association between increasing quartiles of HALP score and serum PSA levels in this research. The HALP score may have the potential to serve as complementary methods for the predictive models in individuals with high PSA level. Given their ease of measurement and non-invasive nature, HALP can play crucial prognostic roles in guiding clinical decisions.

The investigation of innovative biomarkers for predicting survival outcomes has emerged as a trending subject in cancer studies. Previous studies have already proven that some serum biomarkers, such as the neutrophil-to-lymphocyte ratio (NLR), the inflammatory burden index (IBI), the lymphocyte-to-monocyte ratio (LMR), were correlated with survival outcomes in cancer patients (22, 23). Besides, the HALP has attracted increasing attention in lots of studies. By now, HALP has been investigated in numerous research articles assessing outcomes across a wide array of cancers, including PCa, gastrointestinal cancers, lung cancers, among others. And conflicting findings in the prognostic value have been reported in different studies of different type of cancers. In 2015, Chen et al. published the foundational research introducing HALP as a prognostic tool, focusing on survival analysis among patients with gastric carcinoma post-gastrectomy (9). This study indicated that patients with high HALP scores exhibited better overall survival rates (9). Furthermore, HALP showed greater accuracy in prognostic prediction compared to TNM stage alone (9). Then, Guo et al. and their colleagues jointly conducted the initial evaluation of HALP in PCa. They conducted a retrospective study of 82 patients with metastatic PCa after cytoreductive radical prostatectomy in 2019 and found HALP could be an independent prognostic factor (14). HALP scores below 32.4 were observed to be significantly correlated with diminished PSA progression-free survival in both metastatic and oligometastatic PCa subgroups (14). In a 2020 study conducted by Kaya et al. based on patients with BPH and PCa, the HALP score could be used to identify PCa but not suggested to be related with prognosis of patients with PCa (17). A recent reviewed concluded that the HALP score cutoffs for PCa ranged from 32.4 to 51.2, with a median of 41.8 (8). In line with most studies, we found that individuals with lower HALP scores were likely to have reduced overall survival rates.

In fact, all components of HALP, hemoglobin, albumin, lymphocytes, and platelets, can be associated with the occurrence and prognosis of cancer patients, although the specific mechanisms of this association may vary depending on the type, stage of cancer, and individual patient differences. most of cancers are chronic diseases which consume of nutritive substances in the body during the development of the diseases. Hemoglobin levels can be indicative of the oxygen-carrying capacity of blood (24). Low hemoglobin levels are often considered an influential factor in the overall prognosis of cancer patients (25). Higher hemoglobin levels are associated with a lower risk of PCa in a prospective analysis based on data from UK biobank (26). Anemia was associated with poor outcome and increased the risk of death in patients with PCa (24). Low levels of albumin can be associated with malnutrition and cachexia (severe weight loss and muscle wasting) and be related with inflammatory response in the body, which are common in advanced cancer patients (27, 28). Cancer cachexia is often accompanied by cancer-related anemia and hypoalbuminemia, which are indicative of malnutrition (29). Some studies pointed that hypoalbuminemia could be one of possible reasons for the spread of localized PCa and may be associated with biochemical recurrence (30). In addition, lymphopenia is a common and independent prognostic indicator for survival outcomes in individuals with cancers (31). Lymphocytes contribute to immunosurveillance by detecting and eliminating tumor cells. By now, prognostic indicators like the platelet-to-lymphocyte ratio (PLR) and NLR have successfully integrated lymphocyte scores to assess the prognosis of various cancers (32, 33). Platelets have been shown to deeply involve in every stage of tumor development, from the initiation of cancerous growth to its progression, including tumor formation, expansion, and metastasis (34, 35). Platelets have been shown to have the ability to diagnose cancers and detect the development of cancer disease (36). Moreover, several inflammatory markers associated with platelets have been found to be linked to the overall prognosis of individuals of cancer, including PCa patients (37). The HALP score combines all of these biomarkers, which incorporates lymphocytes, hemoglobin, and albumin into its numerator and platelets are factored into the denominator of the calculation. A high HALP score is generally considered a positive biomarker of prognosis for patients with cancer. Same as other studies, our analysis showed a high HALP score is associated with decreased risk and all-cause mortality in participant with high PSA level. Moreover, the HALP score demonstrated superior predictive capability for all-cause mortality in individuals with PSA level when compared to its components.

Despite the availability of numerous detection methods for PCa, the precise risk estimation and overall prognosis for PCa patients remains largely unchanged. Early risk estimation through screening and prognostic tests can lead to timely treatment and improved outcomes for PCa patients. So, identifying high-risk patients with PCa involves considering multiple factors beyond just pathological features. Nutritional status and inflammatory reactions play crucial roles in the progression and prognosis of PCa and should not be overlooked in clinical assessment. The data of the HALP score could be easily obtained from routine blood test. In our study, we have proved the HALP was an easy and powerful biomarker to measure and judge the prognosis of individuals with high PSA level. Therefore, incorporating the HALP score into the assessment scope can be valuable for early risk assessment and prognosis in individuals with high PSA level.

Our study has some limitations. Firstly, our study is a cross-sectional study based on NHANES, so the causal relationship between HALP and the PSA level is not clear. Further longitudinal studies are needed to explore this relationship. Moreover, data from NHANES lacks pathology and imaging results, which could limit the diagnosis for PCa. However, in this study, we used the common diagnostic marker PSA to define the risk of PCa and excluded factors that could influence PSA levels, such as other inflammatory diseases. Thirdly, we utilized a single measurement of the HALP at baseline, but the changes in the HALP over time might be more strongly associated with mortality risk in individuals with high PSA level. Therefore, it suggests the potential importance of longitudinal data and emphasizes the necessity to monitor changes in the HALP over time to better understand its relationship between the HALP score and mortality risk in individuals with high PSA level. Finally, there are potential confounding factors that we have not fully identified or controlled for, which may influence the observed associations.

## Conclusion

Our study demonstrates significant associations between HALP score and serum PSA and all-cause mortality among middle-aged and elderly individuals without PCa. Higher quartiles of HALP score were inversely associated with high PSA level and all-cause mortality, with participants in the highest quartile exhibiting the lowest risk. Furthermore, HALP score exhibited superior predictive ability for all-cause mortality compared to individual HALP components. Notably, the relationship between HALP score and mortality followed a non-linear pattern in participants with high PSA level, with a significant inflection point indicating a plateau phase beyond which no further increase in mortality risk was observed. These findings underscore the potential utility of HALP score as a prognostic marker for mortality risk assessment in individuals with high PSA level. However, further prospective studies are warranted to validate these findings and elucidate the underlying mechanisms driving the observed associations.

## Data Availability

Publicly available datasets were analyzed in this study. This data can be found here: https://wwwn.cdc.gov/nchs/nhanes/.
