# Supplementary material for: Associations of HALP score with serum prostate-specific antigen and mortality in middle-aged and elderly individuals without prostate cancer
Source: Front Oncol. 2024 Sep 20;14:1419310. doi: 10.3389/fonc.2024.1419310 (PMC11449680; doi:10.3389/fonc.2024.1419310)
Supplement: Supplementary file 1 [file DataSheet1.docx]

**Supplementary Materials**

**Associations Between Hemoglobin, Albumin, Lymphocyte and Platelet (HALP) Score, Prostate Cancer Risk, and Mortality Among Middle-aged and Elderly Individuals**

Figure S1. Flowchart of the study

Table S1. Baseline characteristics of participants with men 40 years and older according to PCa risk in NHANES 2001–2010.

Table S2. Linear regression analysis of HALP score with serum PSA levels among men 40 years and older in NHANES 2001–2010.

Table S3. Baseline characteristics of participants with high risk PCa according to all-cause mortality in NHANES 2001–2010.

Table S4. COX regression analysis of HALP score with all-cause mortality stratified by PCa risk (high risk PCa, or low risk PCa) among men 40 years and older after excluding participants who had ccancer history at baseline in NHANES 2001–2010.

Figure S2. Restricted cubic spline (RCS) analysis with multivariate-adjusted associations of HALP score with serum PSA levels and the risk of PCa among men 40 years and older in NHANES 2001–2010.

Figure S3. Kaplan-Meier survival curves for quartiles of HALP components and all-cause mortality among participants with high risk PCa in NHANES 2001–2010.

Table S5. COX regression analysis of HALP components with all-cause mortality among participants with high risk PCa in NHANES 2001–2010.

Figure S4. Restricted cubic spline (RCS) analysis with multivariate-adjusted associations of HALP components with all-cause mortality among participants with high risk PCa in NHANES 2001–2010.

Figure S5. Predictive value of time-dependent ROC assessment of HALP components (A: Hb; B: SAL; C: LYM; and D: PLT) for 3-, 5-, 10-, and 15-year all-cause mortality.

**Materials and methods: Other Covariates**

This section provides a comprehensive definition of potential confounding variables including family poverty income ratio (PIR, ≤1.0, 1.1–3.0, or >3.0), smoking status (never, former, or current smoker), drinking status (nondrinker, former drinker, or current drinker), physical activity (inactive, insufficiently active, or active), healthy eating index (HEI), and Charlson comorbidity index (CCI).

***Family poverty income ratio*** Income was assessed using the poverty income ratio (PIR, the ratio of family income divided by a poverty threshold specific for family size using guidelines from the US Department of Health and Human Services) and categorized as ≤1.0, 1.1-3.0 and >3.0 [1].

***Smoking status*** Never smokers were classified as those who reported smoking <100 cigarettes during their lifetime. Those who smoked >100 cigarettes in their lifetime were considered as current smokers, and those who smoked >100 cigarettes and had quit smoking were considered as former smokers [2].

***Drinking status*** Drinking status was classified as nondrinker, low-to-moderate drinker (<2 drinks/day in men and <1 drink/day in women), or heavy drinker (≥2 drinks/day in men and ≥1 drinks/day in women) [2].

***Physical activity*** Physical activity was categorized as inactive group (no leisure-time physical activity), insufficiently active group (leisure time moderate activity 1–5 times per week with MET ranging from 3 to 6 or leisure-time vigorous activity 1–3 times per week with MET >6), or active group (those who had more leisure-time moderate-or-vigorous activity than above) [3].

***Healthy Eating Index*** The Healthy Eating Index (HEI) is a measure calculated from 24-hour dietary recall data to assess diet quality based on the 2015–2020 Dietary Guidelines for Americans (DGA) [4]. It comprises 13 subgroups, with a total possible score of 100. Nine components evaluate adequacy (higher intakes contribute to a higher score) including total fruits, whole fruits, total vegetables, greens and beans, whole grains, dairy, total protein foods, seafood and plant proteins, and fatty acids. The remaining four components assess moderation (lower intakes yield a higher score), covering refined grains, sodium, added sugars, and saturated fats. The HEI offers a comprehensive framework for understanding dietary patterns and their relationship to health outcomes, with scores reflecting adherence to key dietary recommendations.

***Charlson Comorbidity Index*** The Charlson Comorbidity Index (CCI) is a method used to quantify an individual's overall health status by summing the scores assigned to various diseases [5]. It assumes respondents without reported diseases as healthy, assigning a zero value to unreported conditions. This scoring approach follows the methodology established by Zhao et al. in prior research [6], ensuring consistency in the assessment of comorbidities.

**References**

1. **Services USDoHaH. Poverty Guidelines, Research, and Measurement** [<http://aspe.hhs.gov/POVERTY/index.shtml>.]

2. Qiu Z, Chen X, Geng T, Wan Z, Lu Q, Li L, Zhu K, Zhang X, Liu Y, Lin X *et al*: **Associations of Serum Carotenoids With Risk of Cardiovascular Mortality Among Individuals With Type 2 Diabetes: Results From NHANES**. *Diabetes Care* 2022, **45**(6):1453-1461.

3. Beddhu S, Baird BC, Zitterkoph J, Neilson J, Greene T: **Physical activity and mortality in chronic kidney disease (NHANES III)**. *Clin J Am Soc Nephrol* 2009, **4**(12):1901-1906.

4. Kirkpatrick SI, Reedy J, Krebs-Smith SM, Pannucci TE, Subar AF, Wilson MM, Lerman JL, Tooze JA: **Applications of the Healthy Eating Index for Surveillance, Epidemiology, and Intervention Research: Considerations and Caveats**. *J Acad Nutr Diet* 2018, **118**(9):1603-1621.

5. Kim CY, Sivasundaram L, LaBelle MW, Trivedi NN, Liu RW, Gillespie RJ: **Predicting adverse events, length of stay, and discharge disposition following shoulder arthroplasty: a comparison of the Elixhauser Comorbidity Measure and Charlson Comorbidity Index**. *J Shoulder Elbow Surg* 2018, **27**(10):1748-1755.

6. Zhao H, Pan Y, Wang C, Guo Y, Yao N, Wang H, Li B: **The Effects of Metal Exposures on Charlson Comorbidity Index Using Zero-Inflated Negative Binomial Regression Model: NHANES 2011-2016**. *Biol Trace Elem Res* 2021, **199**(6):2104-2111.

**
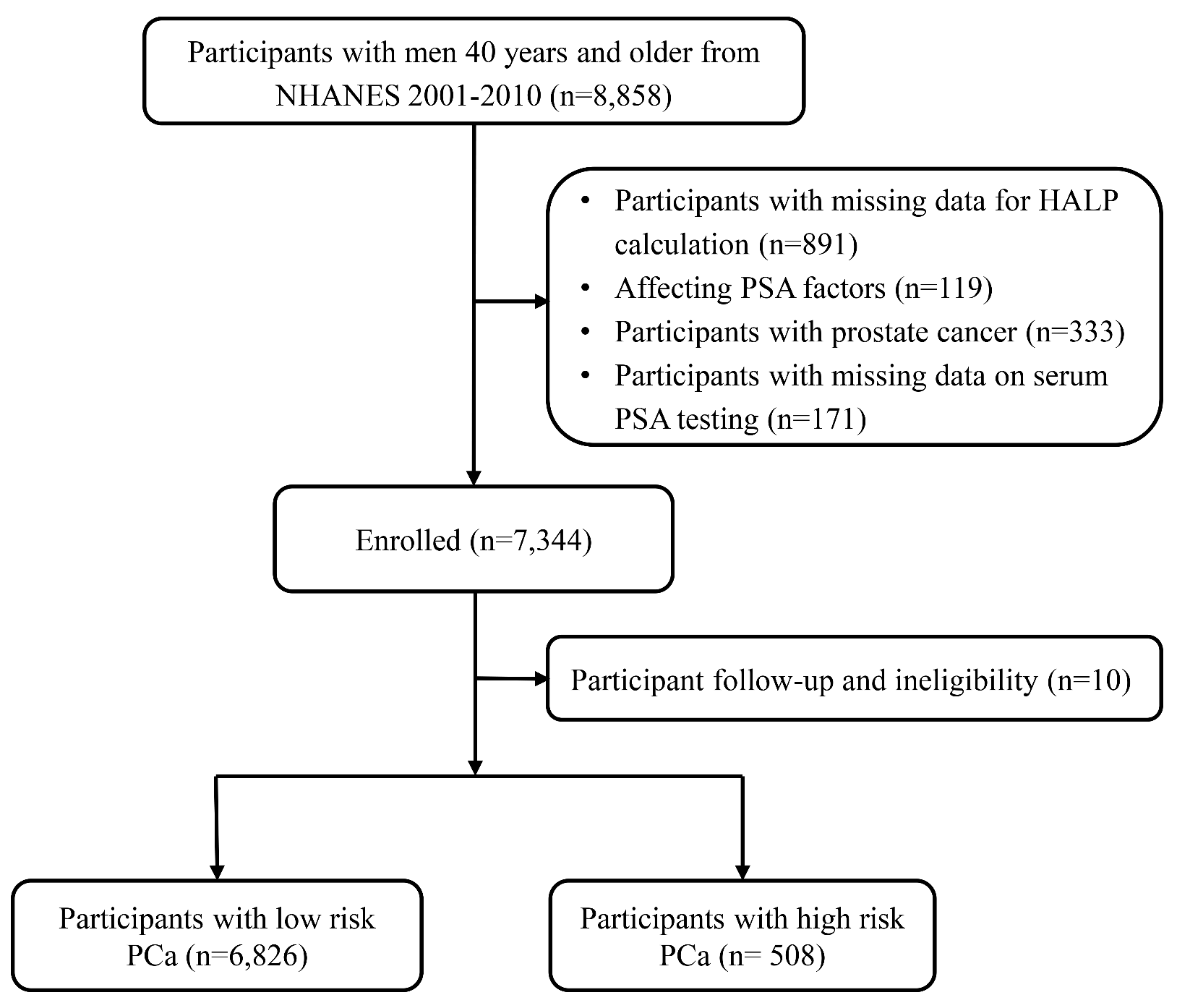
**

**Figure S1.** Flowchart of the study

**Table S1.** Baseline characteristics of middle-aged and elderly individuals without prostate cancer according to PCa risk in NHANES 2001–2010.

| Characteristics | Total | Low PCa risk | High PCa risk | *P* value |
| --- | --- | --- | --- | --- |
| Participants | 7334 | 6826 | 508 |  |
| Age, % |  |  |  | <0.001 |
| 40-59 years | 3730(67.56) | 3648(69.57) | 82(28.28) |  |
| ≥60 years | 3604(32.44) | 3178(30.43) | 426(71.72) |  |
| Race/ethnicity, % |  |  |  | 0.123 |
| Non-Hispanic White | 4012(77.10) | 3732(77.13) | 280(76.47) |  |
| Non-Hispanic Black | 1332(8.85) | 1219(8.72) | 113(11.35) |  |
| Other race | 1990(14.05) | 1875(14.15) | 115(12.18) |  |
| Living status, % |  |  |  | 0.047 |
| Alone | 1997(23.53) | 1827(23.29) | 170(28.40) |  |
| With partners | 5337(76.47) | 4999(76.71) | 338(71.60) |  |
| Education level, % |  |  |  | 0.089 |
| Below high school | 2310(18.56) | 2130(18.32) | 180(23.17) |  |
| High school | 1699(25.24) | 1588(25.40) | 111(22.16) |  |
| Above high school | 3325(56.20) | 3108(56.28) | 217(54.68) |  |
| Family PIR, % |  |  |  | 0.066 |
| ≤1.0 | 1162(9.29) | 1070(9.23) | 92(10.55) |  |
| 1.1–3.0 | 2992(31.26) | 2782(31.00) | 210(36.29) |  |
| >3.0 | 3180(59.45) | 2974(59.77) | 206(53.16) |  |
| Smoking status, % |  |  |  | 0.066 |
| Never smoker | 2752(41.10) | 2559(41.06) | 193(41.92) |  |
| Former smoker | 2893(36.60) | 2672(36.38) | 221(41.07) |  |
| Current smoker | 1689(22.30) | 1595(22.57) | 94(17.00) |  |
| Drinking status, % |  |  |  | 0.040 |
| Nondrinker | 1001(12.21) | 917(11.98) | 84(16.67) |  |
| Low-to-moderate drinker | 5550(76.40) | 5180(76.68) | 370(71.05) |  |
| Heavy drinker | 783(11.39) | 729(11.34) | 54(12.28) |  |
| Body mass index, % |  |  |  | <0.001 |
| <25.0 kg/m^2^ | 1711(21.38) | 1558(20.99) | 153(28.92) |  |
| 25.0-29.9 kg/m^2^ | 3095(42.84) | 2873(42.75) | 222(44.47) |  |
| >29.9 kg/m^2^ | 2528(35.78) | 2395(36.25) | 133(26.61) |  |
| Physical activity, % |  |  |  | 0.013 |
| Inactive | 1981(19.91) | 1818(19.57) | 163(26.61) |  |
| Insufficiently active | 2921(45.75) | 2730(46.05) | 191(39.71) |  |
| Active | 2432(34.34) | 2278(34.37) | 154(33.69) |  |
| HEI-2015 score | 50.12(41.49,59.37) | 49.98(41.38,59.33) | 52.97(43.37,62.02) | 0.008 |
| CCI | 1.00(0.02) | 0.99(0.02) | 1.27(0.09) | 0.004 |
| tPSA, ng/mL | 0.90(0.54,1.60) | 0.84(0.51,1.43) | 6.00(4.78,8.60) | <0.001 |
| fPSA, ng/mL | 0.26(0.17,0.42) | 0.25(0.17,0.39) | 0.98(0.72,1.39) | <0.001 |
| %fPSA, % | 30.00(22.00,38.00) | 30.00(23.00,39.00) | 16.00(12.00,21.00) | <0.001 |
| Hemoglobin, g/dL | 15.20(14.50,16.00) | 15.20(14.50,16.00) | 15.10(14.30,15.90) | 0.092 |
| Albumin, g/L | 43.00(41.00,45.00) | 43.00(41.00,45.00) | 42.00(40.00,44.00) | <0.001 |
| Lymphocyte, 10^3^/μL | 1.90(1.50,2.40) | 1.90(1.60,2.40) | 1.70(1.30,2.20) | <0.001 |
| Platelet, 10^3^/μL | 239.00(203.00,280.00) | 239.00(203.00,280.00) | 240.00(202.00,287.00) | 0.966 |
| HALP score | 52.29(40.30,67.56) | 52.80(40.67,67.99) | 45.48(32.72,57.13) | <0.001 |
| All-cause mortality, % |  |  |  | <0.001 |
| No | 5007(77.72) | 4767(78.80) | 240(56.58) |  |
| Yes | 2327(22.29) | 2059(21.20) | 268(43.42) |  |
| Follow-up time, years | 12.50(10.17,15.50) | 12.58(10.25,15.58) | 11.17(7.25,14.17) | <0.001 |

Abbreviations: PCa, prostate cancer; tPSA, total prostate specific antigen; fPSA, free prostate specific antigen; %fPSA, percent free prostate specific antigen; HEI-2015, Healthy Eating Index 2015; CCI, Charlson Comorbidity Index; HALP, hemoglobin, albumin, lymphocyte and platelet.

Normally distributed continuous variables are described as means ± SEs, and continuous variables without a normal distribution are presented as medians [interquartile ranges]. Categorical variables are presented as numbers (percentages). N reflect the study sample while percentages reflect the survey-weighted data.

**Table S2.** Linear regression analysis of HALP score with serum PSA levels among men 40 years and older in NHANES 2001–2010.

|  | Quartiles of HALP score | | | |  |
| --- | --- | --- | --- | --- | --- |
|  | <39.40 | 39.41-52.03 | 52.04-67.86 | >67.86 | *P* _trend_ |
| tPSA |  |  |  |  |  |
| Crude | 0 [Reference] | -0.407(-0.605,-0.208) | -0.543(-0.753,-0.333) | -0.641(-0.829,-0.453) | <0.001 |
| Model 1 | 0 [Reference] | -0.233(-0.416,-0.050) | -0.355(-0.539,-0.171) | -0.448(-0.625,-0.271) | <0.001 |
| Model 2 | 0 [Reference] | -0.214(-0.397,-0.030) | -0.332(-0.520,-0.144) | -0.428(-0.600,-0.256) | <0.001 |
| fPSA |  |  |  |  |  |
| Crude | 0 [Reference] | -0.068(-0.103,-0.033) | -0.095(-0.131,-0.059) | -0.106(-0.137,-0.075) | <0.001 |
| Model 1 | 0 [Reference] | -0.031(-0.063, 0.002) | -0.054(-0.085,-0.023) | -0.062(-0.092,-0.033) | <0.001 |
| Model 2 | 0 [Reference] | -0.022(-0.054, 0.010) | -0.044(-0.076,-0.012) | -0.049(-0.076,-0.021) | <0.001 |
| %fPSA |  |  |  |  |  |
| Crude | 0 [Reference] | 0.994(0.084,1.904) | 0.994(-0.023,2.011) | 2.15(1.088,3.212) | <0.001 |
| Model 1 | 0 [Reference] | 0.827(-0.081, 1.735) | 0.839(-0.192, 1.871) | 2.032(0.978, 3.085) | <0.001 |
| Model 2 | 0 [Reference] | 1.113(0.172, 2.053) | 1.185(0.124, 2.245) | 2.673(1.591, 3.756) | <0.001 |

Abbreviations: PCa, prostate cancer; HEI-2015, Healthy Eating Index 2015; CCI, Charlson Comorbidity Index; HALP, hemoglobin, albumin, lymphocyte and platelet.

Data are presented as β (95% CI) unless indicated otherwise. Model 1: Adjusted for age (40-59, or ≥60 years), and race/ethnicity (non-Hispanic White, non-Hispanic Black or other race); Model 2: Model 1 + living status (with partners, or alone), education level (below high school, high school, or above high school), family PIR (<1.0, or ≥1.0), BMI (<25.0, 25.0-29.9, or >29.9 kg/m^2^), drinking status (nondrinker, low-to-moderate drinker, or heavy drinker), smoking status (never smoker, former smoker, or current smoker), physical activity (inactive, insufficiently active, or active), HEI (in quartiles), and CCI (continous).

**
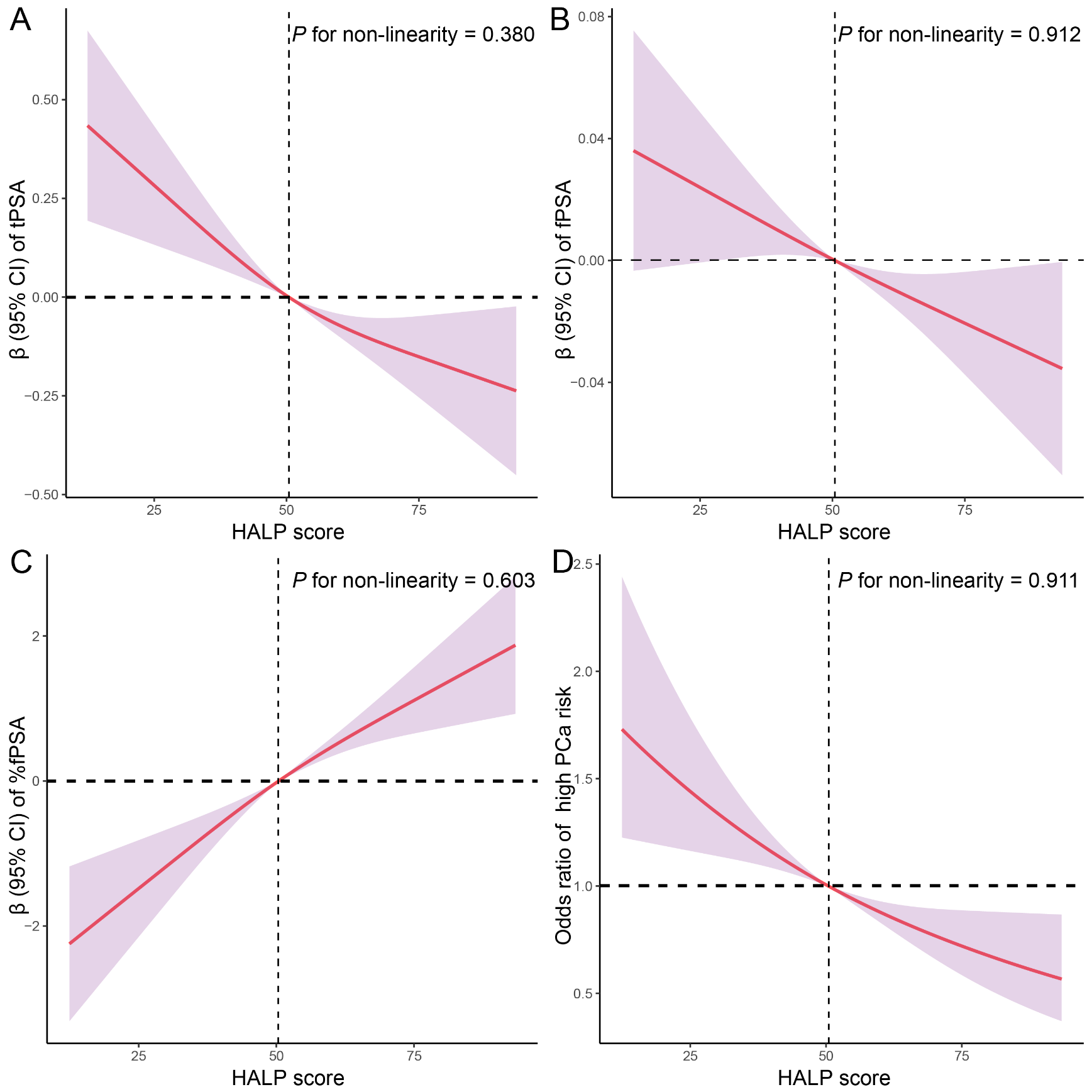
**

**Figure S2.** Restricted cubic spline (RCS) analysis with multivariate-adjusted associations of HALP score with serum PSA levels and the risk of PCa among men 40 years and older in NHANES 2001–2010. Models are adjusted for age (40-59, or ≥60 years), race/ethnicity (non-Hispanic White, non-Hispanic Black or other race), living status (with partners, or alone), education level (below high school, high school, or above high school), family PIR (<1.0, or ≥1.0), BMI (<25.0, 25.0-29.9, or >29.9 kg/m^2^), drinking status (nondrinker, low-to-moderate drinker, or heavy drinker), smoking status (never smoker, former smoker, or current smoker), physical activity (inactive, insufficiently active, or active), HEI (in quartiles), and CCI (continous).

**Table S3.** Baseline characteristics of participants with high risk PCa according to all-cause mortality in NHANES 2001–2010.

| Characteristics | Total (n=508) | All-cause mortality | | *P* value |
| --- | --- | --- | --- | --- |
|  |  | No (n=204) | Yes (n=268) |  |
| Age, % |  |  |  | <0.001 |
| 40-59 years | 82(28.28) | 69(42.90) | 13(9.22) |  |
| ≥60 years | 426(71.72) | 171(57.10) | 255(90.78) |  |
| Race/ethnicity, % |  |  |  | 0.014 |
| Non-Hispanic White | 280(76.47) | 115(73.88) | 165(79.85) |  |
| Non-Hispanic Black | 113(11.35) | 51(10.19) | 62(12.87) |  |
| Other race | 115(12.18) | 74(15.94) | 41(7.29) |  |
| Living status, % |  |  |  | 0.034 |
| Alone | 170(28.40) | 59(23.75) | 111(34.46) |  |
| With partners | 338(71.60) | 181(76.25) | 157(65.54) |  |
| Education level, % |  |  |  | <0.001 |
| Below high school | 180(23.17) | 68(16.39) | 112(32.00) |  |
| High school | 111(22.16) | 50(19.01) | 61(26.26) |  |
| Above high school | 217(54.68) | 122(64.60) | 95(41.74) |  |
| Family PIR, % |  |  |  | <0.001 |
| ≤1.0 | 92(10.55) | 36(9.49) | 56(11.94) |  |
| 1.1–3.0 | 210(36.29) | 80(26.62) | 130(48.88) |  |
| >3.0 | 206(53.16) | 124(63.89) | 82(39.18) |  |
| Smoking status, % |  |  |  | 0.002 |
| Never smoker | 193(41.92) | 111(50.20) | 82(31.15) |  |
| Former smoker | 221(41.07) | 90(33.58) | 131(50.84) |  |
| Current smoker | 94(17.00) | 39(16.22) | 55(18.02) |  |
| Drinking status, % |  |  |  | 0.338 |
| Nondrinker | 84(16.67) | 40(15.47) | 44(18.23) |  |
| Low-to-moderate drinker | 370(71.05) | 169(69.90) | 201(72.55) |  |
| Heavy drinker | 54(12.28) | 31(14.63) | 23(9.22) |  |
| Body mass index, % |  |  |  | 0.882 |
| <25.0 kg/m^2^ | 153(28.92) | 60(28.41) | 93(29.58) |  |
| 25.0-29.9 kg/m^2^ | 222(44.47) | 106(43.96) | 116(45.15) |  |
| >29.9 kg/m^2^ | 133(26.61) | 74(27.64) | 59(25.27) |  |
| Physical activity, % |  |  |  | <0.001 |
| Inactive | 163(26.61) | 53(17.79) | 110(38.10) |  |
| Insufficiently active | 191(39.71) | 102(43.45) | 89(34.83) |  |
| Active | 154(33.69) | 85(38.76) | 69(27.08) |  |
| HEI-2015 score | 52.97(43.37,62.02) | 52.97(43.38,62.33) | 53.27(43.13,61.36) | 0.953 |
| CCI | 1.27(0.09) | 1.04(0.13) | 1.56(0.13) | 0.013 |
| tPSA, ng/mL | 6.00(4.78,8.60) | 5.67(4.77, 7.59) | 6.70(4.80,10.00) | 0.025 |
| fPSA, ng/mL | 0.98(0.72,1.39) | 0.93(0.67,1.27) | 1.07(0.79,1.54) | 0.012 |
| %fPSA, % | 16.00(12.00,21.00) | 16.00(12.00,21.00) | 15.00(12.00,21.00) | 0.789 |
| Hemoglobin, g/dL | 15.10(14.30,15.90) | 15.40(14.70,15.90) | 14.70(13.80,15.70) | <0.001 |
| Albumin, g/L | 42.00(40.00,44.00) | 42.00(40.00,43.00) | 42.00(40.00,44.00) | 0.585 |
| Lymphocyte, 103/μL | 1.70(1.30,2.20) | 1.70(1.40,2.20) | 1.50(1.20,2.10) | 0.019 |
| Platelet, 103/μL | 240.00(202.00,287.00) | 244.00(210.00,287.00) | 234.00(191.00,285.00) | 0.260 |
| HALP score | 45.48(32.72,57.13) | 47.16(35.50,60.17) | 43.40(30.45,54.02) | 0.015 |

Abbreviations: PCa, prostate cancer; tPSA, total prostate specific antigen; fPSA, free prostate specific antigen; %fPSA, percent free prostate specific antigen; HEI-2015, Healthy Eating Index 2015; CCI, Charlson Comorbidity Index; HALP, hemoglobin, albumin, lymphocyte and platelet.

Normally distributed continuous variables are described as means ± SEs, and continuous variables without a normal distribution are presented as medians [interquartile ranges]. Categorical variables are presented as numbers (percentages). N reflect the study sample while percentages reflect the survey-weighted data.

**Table S4.** COX regression analysis of HALP score with all-cause mortality stratified by PCa risk (high risk PCa, or low risk PCa) among men 40 years and older after excluding participants who had ccancer history at baseline in NHANES 2001–2010.

|  | Crude | |  | Model 1 | |  | Model 2 | |
| --- | --- | --- | --- | --- | --- | --- | --- | --- |
|  | HR (95% CI) | *P* value |  | HR (95% CI) | *P* value |  | HR (95% CI) | *P* value |
| High risk PCa (n = 435) | |  |  |  |  |  |  |  |
| Quartiles of HALP levels | |  |  |  |  |  |  |  |
| Q1 | 1 [Reference] |  |  | 1 [Reference] |  |  | 1 [Reference] |  |
| Q2 | 0.725(0.506,1.040) | 0.08 |  | 0.730(0.508,1.051) | 0.091 |  | 0.935(0.640,1.365) | 0.727 |
| Q3 | 0.722(0.502,1.036) | 0.077 |  | 0.701(0.487,1.010) | 0.056 |  | 0.861(0.582,1.275) | 0.456 |
| Q4 | 0.543(0.370,0.796) | 0.002 |  | 0.607(0.414,0.891) | 0.011 |  | 0.563(0.376,0.843) | 0.005 |
| *P* for trend |  | 0.003 |  |  | 0.012 |  |  | 0.006 |
| Low risk PCa (n = 6,238) | |  |  |  |  |  |  |  |
| Quartiles of HALP levels | |  |  |  |  |  |  |  |
| Q1 | 1 [Reference] |  |  | 1 [Reference] |  |  | 1 [Reference] |  |
| Q2 | 0.627(0.552,0.713) | <0.001 |  | 0.731(0.642,0.831) | <0.001 |  | 0.769(0.675,0.875) | <0.001 |
| Q3 | 0.606(0.532,0.690) | <0.001 |  | 0.709(0.622,0.808) | <0.001 |  | 0.734(0.642,0.838) | <0.001 |
| Q4 | 0.692(0.609,0.786) | <0.001 |  | 0.842(0.741,0.957) | 0.009 |  | 0.830(0.728,0.946) | 0.005 |
| *P* for trend |  | <0.001 |  |  | 0.002 |  |  | 0.002 |

Abbreviations: PCa, prostate cancer; HEI-2015, Healthy Eating Index 2015; CCI, Charlson Comorbidity Index; HALP, hemoglobin, albumin, lymphocyte and platelet.

Data are presented as OR (95% CI) unless indicated otherwise. Model 1: Adjusted for age (40-59, or ≥60 years), and race/ethnicity (non-Hispanic White, non-Hispanic Black or other race); Model 2: Model 1 + living status (with partners, or alone), education level (below high school, high school, or above high school), family PIR (<1.0, or ≥1.0), BMI (<25.0, 25.0-29.9, or >29.9 kg/m^2^), drinking status (nondrinker, low-to-moderate drinker, or heavy drinker), smoking status (never smoker, former smoker, or current smoker), physical activity (inactive, insufficiently active, or active), HEI (in quartiles), and CCI (continous).


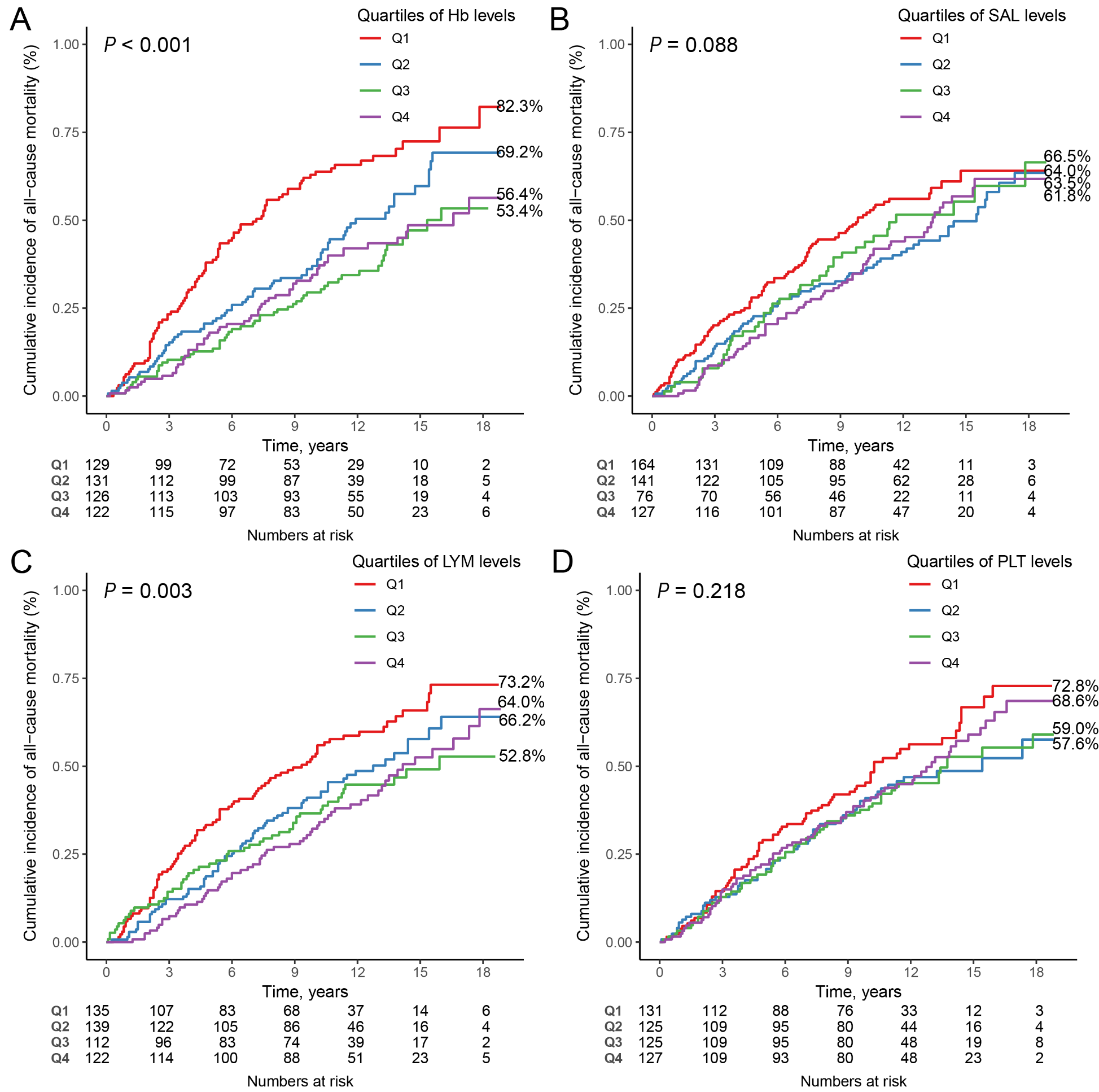


**Figure S3**. Kaplan-Meier survival curves for quartiles of HALP components (A: Hb; B: SAL; C: LYM; and D: PLT) and all-cause mortality among participants with high risk PCa in NHANES 2001–2010.

**Table S5.** COX regression analysis of HALP components with all-cause mortality among participants with high risk PCa in NHANES 2001–2010.

|  | Quartiles of HALP components | | | |  |
| --- | --- | --- | --- | --- | --- |
|  | HR | HR (95% CI) | HR (95% CI) | HR (95% CI) | *P* _trend_ |
| Hemoglobin |  |  |  |  |  |
| Crude | 1 [Reference] | 0.593(0.433,0.811) | 0.404(0.287,0.570) | 0.452(0.323,0.632) | <0.001 |
| Model 1 | 1 [Reference] | 0.641(0.467,0.879) | 0.486(0.341,0.691) | 0.513(0.361,0.729) | <0.001 |
| Model 2 | 1 [Reference] | 0.701(0.504,0.974) | 0.522(0.361,0.755) | 0.649(0.446,0.943) | 0.006 |
| Albumin |  |  |  |  |  |
| Crude | 1 [Reference] | 0.696(0.509,0.950) | 0.802(0.553,1.161) | 0.726(0.529,0.997) | 0.087 |
| Model 1 | 1 [Reference] | 0.730(0.533,1.000) | 0.842(0.580,1.221) | 0.782(0.566,1.080) | 0.21 |
| Model 2 | 1 [Reference] | 0.787(0.567,1.094) | 0.929(0.632,1.365) | 0.893(0.637,1.251) | 0.697 |
| Lymphocyte |  |  |  |  |  |
| Crude | 1 [Reference] | 0.707(0.517,0.967) | 0.605(0.428,0.856) | 0.579(0.416,0.807) | <0.001 |
| Model 1 | 1 [Reference] | 0.726(0.529,0.994) | 0.694(0.490,0.983) | 0.661(0.471,0.927) | 0.017 |
| Model 2 | 1 [Reference] | 0.740(0.534,1.026) | 0.705(0.491,1.011) | 0.591(0.415,0.843) | 0.004 |
| Platelet |  |  |  |  |  |
| Crude | 1 [Reference] | 0.738(0.526,1.035) | 0.734(0.524,1.027) | 0.832(0.601,1.152) | 0.290 |
| Model 1 | 1 [Reference] | 0.776(0.552, 1.090) | 0.784(0.560, 1.098) | 0.953(0.688, 1.321) | 0.768 |
| Model 2 | 1 [Reference] | 0.785(0.552,1.118) | 0.839(0.592,1.189) | 0.863(0.613,1.214) | 0.478 |

Abbreviations: PCa, prostate cancer; HEI-2015, Healthy Eating Index 2015; CCI, Charlson Comorbidity Index; HALP, hemoglobin, albumin, lymphocyte and platelet.

Data are presented as OR (95% CI) unless indicated otherwise. Model 1: Adjusted for age (40-59, or ≥60 years), and race/ethnicity (non-Hispanic White, non-Hispanic Black or other race); Model 2: Model 1 + living status (with partners, or alone), education level (below high school, high school, or above high school), family PIR (<1.0, or ≥1.0), BMI (<25.0, 25.0-29.9, or >29.9 kg/m^2^), drinking status (nondrinker, low-to-moderate drinker, or heavy drinker), smoking status (never smoker, former smoker, or current smoker), physical activity (inactive, insufficiently active, or active), HEI (in quartiles), and CCI (continous).

**
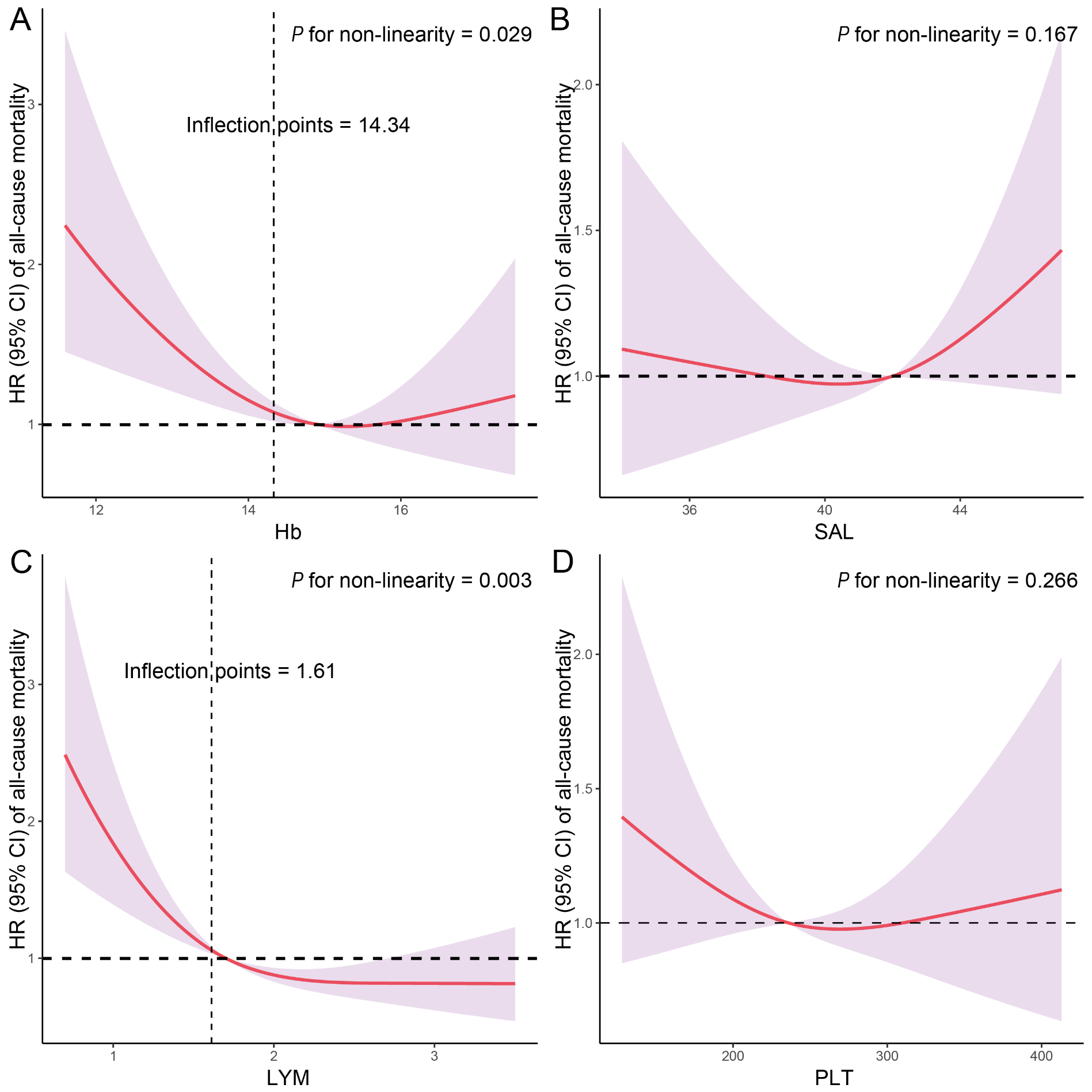
**

**Figure S4.** Restricted cubic spline (RCS) analysis with multivariate-adjusted associations of HALP components (A: Hb; B: SAL; C: LYM; and D: PLT) with all-cause mortality among participants with high risk PCa in NHANES 2001–2010. Models are adjusted for age (40-59, or ≥60 years), race/ethnicity (non-Hispanic White, non-Hispanic Black or other race), living status (with partners, or alone), education level (below high school, high school, or above high school), family PIR (<1.0, or ≥1.0), BMI (<25.0, 25.0-29.9, or >29.9 kg/m^2^), drinking status (nondrinker, low-to-moderate drinker, or heavy drinker), smoking status (never smoker, former smoker, or current smoker), physical activity (inactive, insufficiently active, or active), HEI (in quartiles), and CCI (continous).


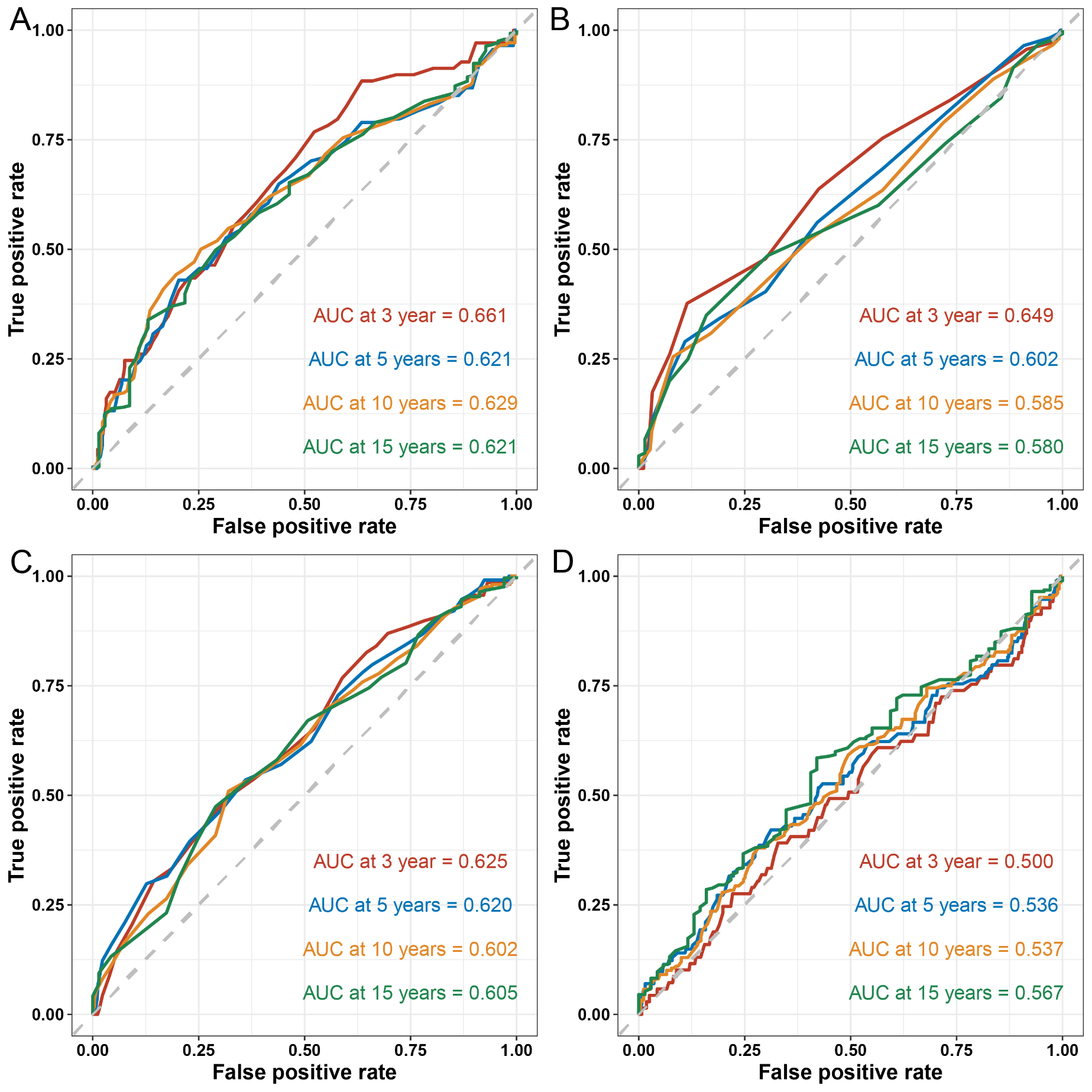


**Figure S5.** Predictive value of time-dependent ROC assessment of HALP components (A: Hb; B: SAL; C: LYM; and D: PLT) for 3-, 5-, 10-, and 15-year all-cause mortality.
